# Anti-inflammatory effect of *Vaccinium oldhamii* stems through inhibition of NF-κB and MAPK/ATF2 signaling activation in LPS-stimulated RAW264*.*7 cells

**DOI:** 10.1186/s12906-019-2720-4

**Published:** 2019-11-04

**Authors:** Ha Na Kim, Jueng Kyu Baek, Su Bin Park, Jeong Dong Kim, Ho-Jun Son, Gwang Hun Park, Hyun Ji Eo, Jae Ho Park, Hyuk-Sang Jung, Jin Boo Jeong

**Affiliations:** 1 Department of Medicinal Plant Resources, Andong National University, Andong, 36729 Republic of Korea; 2 Forest Medicinal Resources Research Center, National Institute of Forest Science, Yeongju, 36040 Republic of Korea; 3 Department of Pharmaceutical Science, Jungwon University, Geosan, Chungbuk 28024 Republic of Korea; 4 Department of Anatomy, College of Korean Medicine, Kyung Hee University, Seoul, 02447 Republic of Korea; 5 Insititute of Agricultural Science and Technology, Andong National University, Andong, 36729 Republic of Korea

**Keywords:** Anti-inflammation, Anti-osteoclastogenesis, Inflammatory diseases, *Vaccinium oldhamii*

## Abstract

**Background:**

*Vaccinium oldhamii* (*V. oldhamii*) has been reported to exert a variety of the pharmacological properties such as anti-oxidant activity, anti-cancer activity, and inhibitory activity of α-amylase and acetylcholinesterase. However, the anti-inflammatory activity of *V. oldhamii* has not been studied. In this study, we aimed to investigate anti-inflammatory activity of the stem extracts from *V. oldhamii*, and to elucidate the potential mechanisms in LPS-stimulated RAW264.7 cells.

**Methods:**

Cell viability was evaluated by MTT assay. The determination of NO and PGE2 production was performed using Griess reagent and Prostaglandin E_2_ ELISA Kit, respectively. The change of mRNA or protein level was evaluated by RT-PCR and Western blot.

**Results:**

Among VOS, VOL and VOF, the inhibitory effect of NO and PGE_2_ production induced by LPS was highest in VOS treatment. Thus, VOS was selected for the further study. VOS dose-dependently blocked LPS-induced NO and PGE_2_ production by inhibiting iNOS and COX-2 expression, respectively. VOS inhibited the expression of pro-inflammatory cytokines such as IL-1β, IL-6 and TNF-α. In addition, VOS suppressed TRAP activity and attenuated the expression of the osteoclast-specific genes such as NFATc1, c-FOS, TRAP, MMP-9, cathepsin K, CA2, OSCAR and ATPv06d2. VOS inhibited LPS-induced NF-κB signaling activation through blocking IκB-α degradation and p65 nuclear accumulation. VOS inhibited MAPK signaling activation by attenuating the phosphorylation of ERK1/2, p38 and JNK. Furthermore, VOS inhibited ATF2 phosphorylation and blocked ATF2 nuclear accumulation.

**Conclusions:**

These results indicate that VOS may exert anti-inflammatory activity by inhibiting NF-κB and MAPK/ATF2 signaling. From these findings, VOS has potential to be a candidate for the development of chemopreventive or therapeutic agents for the inflammatory diseases.

## Background

Although inflammation is known to be a defense mechanism against noxious stimuli, abnormal inflammatory response causes a variety of human diseases such as obesity [1], cardiovascular [2] and neurodegenerative disease [3], cancer [4] and osteoporosis [5].

Of the various inflammatory mediators, nitric oxide (NO) contributes to anti-inflammatory activity in normal physiological conditions [6], but excessive NO production is thought to cause chronic inflammation in abnormal situation, which indicates that NO is a major molecule that plays a key role in the pathogenesis of inflammatory disorders [6]. Since inducible nitric oxide synthase (iNOS) is involved in the synthesis of NO, inhibition of iNOS expression has been regarded as an important molecular target for anti-inflammatory action [7, 8]. In addition to NO, prostaglandin E_2_ (PGE_2_) produced by cyclooxygenase-2 (COX-2) is also associated with the progression of the inflammatory diseases induced by chronic inflammation [9]. Therefore, suppression of NO and PGE_2_ production through inhibition of iNOS and COX-2 expression, respectively has been thought to be important targets for the treatment of inflammatory diseases [6, 10]. The inflammatory mediators such as NO, PGE_2_, iNOS, COX-2 and IL-1β have been known to be closely related to the pathogenesis of osteoporosis in the human inflammatory diseases [11].

For the evaluating the pharmacological activity of plants, the choice of plant species has been usually determined by the fact that it is already used for some purpose. *Vaccinium oldhamii* Miquel (*V. oldhamii*) native in Korea has been used to treat gonorrhea, vomiting, diarrhea, eruption and inflammation [12]. The fruit of *V. oldhamii* has been reported to exert anti-oxidant and anti-cancer activity [13]. In addition, *V. oldhamii* inhibits α-amylase and acetylcholinesterase [12, 14]. The fruit of *V. oldhamii* is considered to be an important resource for the development of new blueberry cultivars [13] because it has higher antioxidant activity than blueberries [15]. The contents of anthocyanin and polyphenol from the fruit of *V. oldhamii* have been reported to be higher than those of southern highbush blueberry and northern highbush blueberry [13]. In addition, *V. oldhami* leaves have been reported to inhibit NO production in LPS-stimulated RAW264.7 cells [16].

Although the anti-inflammatory activity of *V. oldhamii* have been reported, it is still insufficient. Thus, in this study, we compared the anti-inflammatory activity of the plant parts of *V. oldhamii* such as stems, leaves and fruits. In addition, we investigated the mechanism of action on anti-inflammatory activity of the stems with the highest anti-inflammatory activity.

## Materials and methods

### Materials

Dulbecco’s Modified Eagle medium (DMEM)/F-12 1:1 Modified medium (DMEM/F-12) for cell culture was purchased from Lonza (Walkersville, MD, USA). 3-(4,5-dimethylthiazol-2-yl)-2,5-diphenyltetrazolium bromide (MTT), 2,2-diphenyl-1-picrylhydrazyl (DPPH), tolfenamic acid (TA), tartrate-resistant acid phosphatase (TRAP) solution and lipopolysaccharide (LPS) for inflammation induction was purchased from Sigma Aldrich (St. Louis, MO, USA). Antibodies against iNOS (#13120), COX-2 (#12282), IκB-α (#4814), p65 (#8242), phospho-ERK1/2 (#4377), ERK1/2 (#9102), phospho-p38 (#4511), p38 (#9212), phospho-JNK (#4668), JNK (#9258), p-ATF2 (#9221), ATF2 (#35031) and β-actin (#5125) were purchased from Cell Signaling (Bervely, MA, USA). Antibodies such as NFATc1 (#556602) and c-Fos (SC-52) were purchased from BD Pharmingen (San Diego, CA, USA) and Santa Cruz Biotechnology (Santa Cruz, CA, USA), respectively.

### Preparation of extracts

The extraction of *V. oldhami* (VO) was carried out according to the literatures with some modification [13, 16]. VO (voucher number: Jeong 201,802 (ANH)) was generously provided from Forest Medicinal Resources Research Center, National Institute of Forest Science, Yongju, Korea. VO was formally identified by Ho-Jun Son a researcher of Forest Medicinal Resources Research Center, Korea. Five grams of the stems, leaves and fruits from VO were extracted with 100 ml of 70% ethanol for 72 h under stirring at room temperature. After 72 h, the ethanol extracts were filtered and concentrated to approximately 30 ml volume using a vacuum evaporator and then freeze-dried. The ethanol extracts from the stems (VOS), leaves (VOL) or fruits (VOF) of VO were kept in a refrigerator until use.

### Analysis of extracts

The analysis of anti-inflammatory compounds from VOS was performed using GC/MS and HPLC. In GC/MS analysis, Agilent 6890 GC interfaced to an Agilent 5973 MS equipped with an EI source and autoinjector (Agilent Technologies, Santa Clara, CA, USA) was used. The GC system was equipped with a HP-5 column (30.0 m × 0.25 mm × 0.25 μm). The oven temperature was 70 °C (5 min) and raised to 290 °C (5 min) at 5 °C/min, and injection volume was 1 μl. The injection was performed in the split mode adjusted to 1:5. The carrier gas was helium at 1.0 ml/min. Inlet, source and quadrupole temperatures were set at 290, 230 and 190 °C, respectively. For MS detection, the electron ionization mode with an ionization energy of 70 eV was used with a mass range at m/z 50–550. Agilent ChemStation software was used for data processing. Anti-inflammatory compounds from VOS were identified by mass fragmentation patterns compared by using Wiley Spectral library search program. In HPLC analysis, Waters 1525 system with a Waters 2487-dual λ absorbance detector was used. The column was equipped with the SUNFIRE C18 column (250 mm × 4.6 mm). The binary mobile phase consisted of 14% methanol (solvent A) and 86% water (solvent B, pH 3.1). The flow rate was kept constant at 1.0 ml/min for a total run time of 60 min. The injection volume of the extract was 5 μl. The elution was monitored at 280 nm. Anti-inflammatory compounds from VOS were identified by the chromatogram of the analytical standards such as (+)-catechin, (−)-epicatechin, proanthocyanidin A2 and cinnamtannin.

### DPPH radical scavenging assay

DPPH radical scavenging assay was applied to evaluate anti-oxidant activity of VOS, VOL or VOF. DPPH radical scavenging assay was carried out according to the literatures with some modification [17, 18]. Briefly, 152 μl of DPPH solution (1 mM DPPH in 95% ethanol) was added with 8 μl of VOS, VOL or VOF containing different concentrations (25 and 50 μg/ml) in 96-well plate. The mixtures were reacted for 30 min in the dark at 37 °C. After reaction, the absorbance was measured at a wavelength of 517 nm using UV/Visible spectrophotometer (Human Cop., Xma-3000PC, Seoul, Korea).

### Determination of the contents of total phenolic compounds

The contents of total phenolic compounds were measured using the Folin-Ciocalteu assay [18]. Briefly, 0.5 ml of VOS (50 mg/ml), VOL (50 mg/ml) or VOF (50 mg/ml) in 1 ml of distilled water was mixed with 0.5 ml of 2 N Folin-Ciocalteu reagent for 5 min, and then added 2 ml of 7% (w/v) sodium carbonate. The mixtures were incubated for 90 min at room temperature. After 90 min, the absorbance was measured a wavelength of 750 nm using UV/Visible spectrophotometer (Human Cop., Xma-3000PC, Seoul, Korea).

### Cell culture and treatment

Mouse macrophage cell line, RAW264.7 has long been used for the evaluating anti-inflammatory activity. Thus, RAW264.7 cells were used in this study. RAW264.7 cells was purchased from Korean Cell Line Bank (Seoul, Korea) and maintained at 37 °C under a humidified atmosphere of 5% CO_2_ using DMEM/F-12 media containing 10% fetal bovine serum (FBS), 100 U/ml penicillin and 100 μg/ml streptomycin. VOS, VOL or VOF was dissolved in dimethyl sulfoxide (DMSO) and treated to cells. DMSO was used as a control and the final DMSO concentration did not exceed 0.1% (v/v).

### Cell viability assay

MTT assay was applied to evaluate cytotoxicity of VOS, VOL or VOF. MTT assay was carried out according to the literatures with some modification [16]. Briefly, VOS, VOL or VOF was treated to the cells cultured on a 96-well plate at a density of 3 × 10^3^ cells/well for 24 h. Then, the cells and incubated for 2 h after adding 50 μl of MTT solution (1 mg/ml). Then, cell culture supernatants were removed and DMSO was added to the cells for dissolving the resulting crystals. The formation of formazan was measured by reading absorbance at a wavelength of 570 nm using UV/Visible spectrophotometer (Human Cop., Xma-3000PC, Seoul, Korea).

### Determination of NO, PGE_2_, IL-1β, IL-6 and TNF-α

Determining NO production was performed using Griess assay according to the literatures with some modification [16]. Briefly, VOS, VOL or VOF was pretreated to the cells cultured on a 12-well plate at a density of 1 × 10^5^ cells/well for 6 h. After 6 h, LPS (1 μg/ml) was co-treated to the cells for 18 h to induce inflammatory response. Then, 100 μl of the cell culture supernatants was mixed with 100 μl of Griess reagent (Sigma Aldrich), reacted at room temperature for 15 min, the absorbance was measured at 540 nm using UV/Visible spectrophotometer (Human Cop., Xma-3000PC, Seoul, Korea). The level of PGE_2_, IL-1β, IL-6 or TNF-α levels were measured accordingly with the manufacturer’s protocols of Prostaglandin E_2_ ELISA Kit (Cayman Chemical, Ann Arbor, MI, USA), Mouse IL-1β ELISA Kit (Invitrogen, Carlsbad, CA, USA), IL-6 (Mouse) ELISA Kit (Cayman Chemical), TNF-α (Mouse) ELISA Kit (Cayman Chemical).

### TRAP assay

TRAP assay was carried out according to the literatures with some modification [5]. To differentiate the effect of VOS on osteoclastogenesis, RAW 264.7 cells at 5 × 10^3^ cells per well were seeded on a 96-well plate with RANKL (100 ng/ml) and various concentrations of VOS. Five days later, cells were fixed using a 10% formalin solution and stained for TRAP according to the manufacturer’s protocol. The stained cells were imaged using an inverted microscope (100×) and measured using Image J software (National Institutes of Health, Bethesda, MD, USA). TRAP activity was determined in the supernatants collected from wells using a TRAP solution (Pnpp in 0.5 M acetate, dissolved with tartrate acid solution).

### Isolation of nucleus fraction

Nuclear fractions of cells were extracted using a nuclear extract kit (Active Motif, Carlsbad, CA, USA) according to the manufacturer’s protocols. Briefly, RAW264.7 cells were collected with cold 1 × hypotonic buffer and reacted at 4 °C for 15 min. Then, detergent was added and vortexed for 10 s. The cells were centrifuged at 14,000 g for 1 min at 4 °C and the cell pellets were used for nuclear fraction collection. Nuclear fractions from the cell pellets were extracted using complete lysis buffer by the incubation at 4 °C for 30 min under shaking. After 30 min, nuclear fractions from the cell pellets were centrifuged at 14,000 g for 10 min at 4 °C, and the supernatants (nuclear fraction) were stored at − 80 °C for further analysis.

### SDS-PAGE and Western blot

After treatment, the cells were washed twice with cold 1 × phosphate-buffered saline (PBS), and the cellular proteins were extracted using radioimmunoprecipitation assay (RIPA) buffer (Boston Bio Products, Ashland, MA, USA) supplemented with protease inhibitor cocktail (Sigma-Aldrich) and phosphatase inhibitor cocktail (Sigma-Aldrich). The concentration of the proteins extracted from the cells was quantified using BCA protein assay (Thermo Fisher Scientific, Waltham, MA USA). The equal protein (30 μg/well) was separated on SDS-PAGE and transferred to PVDF membrane (Bio-Rad Laboratories, Inc., Hercules, CA, USA). The PVDF membranes were blocked with 5% non-fat dry milk in Tris-buffered saline containing 0.05% Tween 20 (TBS-T) by stirring at room temperature for 1 h and then incubated with specific primary antibodies (1:1000) in 5% non-fat dry milk in 0.05% TBS-T at 4 °C for 16 h. After 16 h, the PVDF membranes were washed three times for 5 min with 0.05% TBS-T, and then incubated with horse radish peroxidase-conjugated immunoglobulin G (1:1000) for 1 h at room temperature. Chemiluminescence was detected with ECL Western blotting substrate (Amersham Biosciences, Piscataway, NJ, USA) and visualized in Polaroid film. The density of Western blot bands was calculated using the software UN-SCAN-IT gel version 5.1 (Silk Scientific Inc. Orem, UT, USA).

### Reverse transcriptase-polymerase chain reaction (RT-PCR)

After treatment, total RNA was extracted from the cells using a RNeasy Mini Kit (Qiagen, Valencia, CA, USA) and 1 μg of total RNA was synthesized using a Verso cDNA Kit (Thermo Scientific, Pittsburgh, PA, USA) according to the manufacturer’s protocol. PCR was performed using PCR Master Mix Kit (Promega, Madison, WI, USA). The primer sequences used in this study were shown in Table 1. The PCR results were visualized using agarose gel electrophoresis. PCR reaction conditions were used: 1 cycle of (3 min at 94 °C for denaturation), 30 cycles of (30 s at 94 °C for denaturation, 30 s at 60 °C for annealing, and 30 s at 72 °C for elongation), and 1 cycle of (5 min for extension at 72 °C). The density of mRNA bands was calculated using the software UN-SCAN-IT gel version 5.1 (Silk Scientific Inc. Orem, UT, USA).

**Table 1:** The primer sequences used in this study

Primers	Sequences
iNOS	Forward 5′-ttgtgcatcgacctaggctggaa-3′ Reverse 5′-gacctttcgcattagcatggaagc-3′
COX-2	Forward 5′-gtactggctcatgctggacga-3′ Reverse 5′-caccatacactgccaggtcagcaa-3′
IL-1β	Forward 5′-ggcaggcagtatcactcatt-3′ Reverse 5′-cccaaggccacaggtattt-3′
IL-6	Forward 5′-gaggataccactcccaacagacc-3′ Reverse 5′-aagtgcatcatcgttgttcataca-3′
TNF-α	Forward 5′-tggaactggcagaagaggca-3′ Reverse 5′-tgctcctccacttggtggtt-3′
TRAP	Forward 5′-acttccccagcccttactaccg-3′ Reverse 5′-tcagcacatagcccacaccg-3’
NFATc1	Forward 5’-tgctcctcctcctgctgctc-3′ Reverse 5′-cgtcttccacctccacgtcg-3’
c-Fos	Forward 5’-atgggctctcctgtcaacac-3′ Reverse 5′-ggctgccaaaataaactcca-3’
MMP-9	Forward 5’-cgacttttgtggtcttcccc-3′ Reverse 5′-tgaaggtttggaatcgaccc-3’
CTK	Forward 5’-aggcggctatatgaccactg-3′ Reverse 5′-ccgagccaagagagcatatc-3’
CA2	Forward 5’-ctctcaggacaatgcagtgctga-3′ Reverse 5′-atccaggtcacacattccagca-3’
OSCAR	Forward 5’-ctgctggtaacggatcagctccccaga-3′ Reverse 5′-ccaaggagccagaaccttcgaaact-3’
ATP6v0d2	Forward 5’-atggggccttgcaaaagaaatctg-3′ Reverse 5′-cgacagcgtcaaacaaaggcttgta-3’
GAPDH	Forward 5′-ggactgtggtcatgagcccttcca-3′ Reverse 5′-actcacggcaaattcaacggcac-3′

### Transient transfection and luciferase activity

Transient transfection for luciferase activity was performed using the PolyJet DNA transfection reagent (SignaGen Laboratories, Ijamsville, MD, USA). Cells cultured on 12-well plates at a density of 2 × 10^5^ cells/well were treated with plasmid mixtures containing 1 μg of the NF-κB luciferase constructs (Addgene, Cambridge, MA, USA) and 0.1 μg of pRL-null vector, and then cultured for 24 h. After 24 h, VOS was pretreated to the cells for 6 h, and then LPS (1 μg/ml) was co-treated to the cells for 18 h. After treatment, the cells were then harvested in 1 × luciferase lysis buffer, and luciferase activity was normalized to the pRL-null luciferase activity using a dual-luciferase assay kit (Promega, Madison, WI, USA).

### Statistical analysis

All the data are shown as mean ± SD (standard deviation). Statistical analysis was performed with one-way ANOVA followed by Dunnett’s test. Differences with *P or ^#^*P* < 0.05 were considered statistically significant.

## Results

### Analysis of bioactive components from VOS

**Fig. 1:**
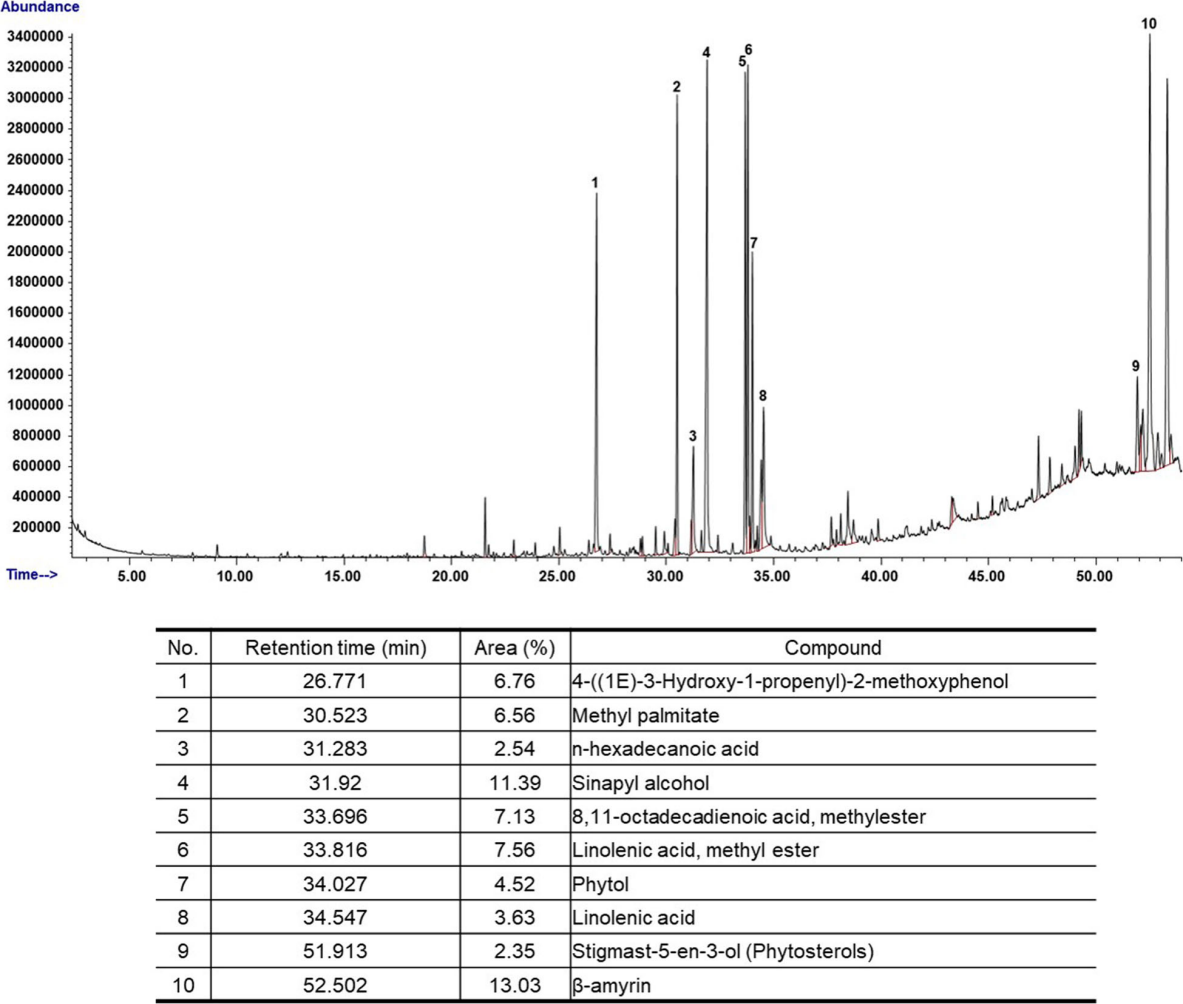
Chromatography of GC/MS analysis of VOS. The major compounds from VOS were analyzed using GC/MS as described in Materials and methods

**Fig. 2:**
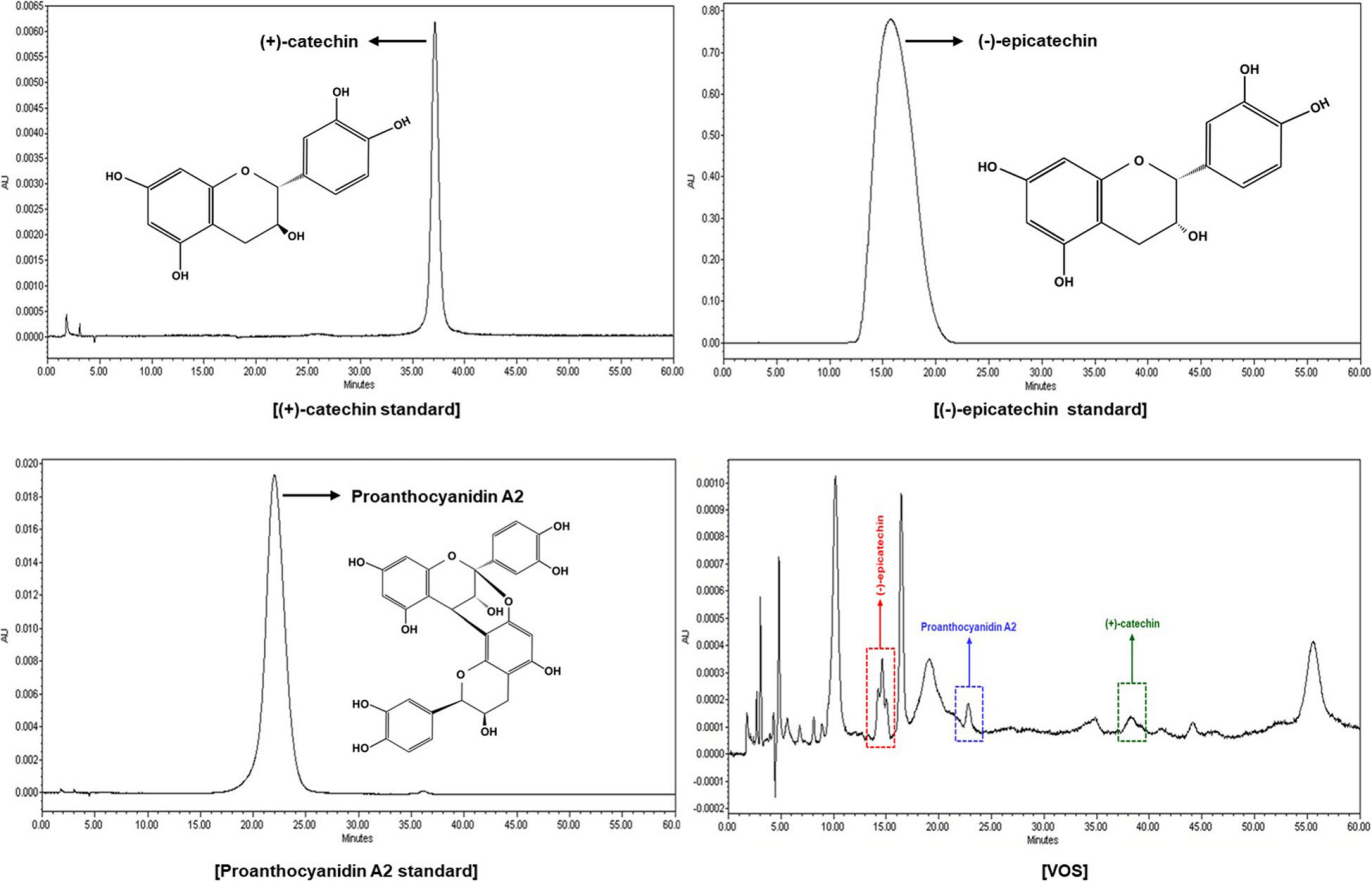
Chromatography of HPLC analysis of VOS. The major compounds from VOS were analyzed using HPLC as described in Materials and methods

To analyze the potential medicinal compounds with anti-inflammatory activity from VOS, we performed GC/MS analysis and HPLC of VOS. As shown in Fig. 1, VOS was analyzed to contain ten compounds such as 4-((1E)-3-Hydroxy-1-propenyl)-2-methoxyphenol, methyl palmitate, n-hexadecanoic acid, sinapyl alcohol, 8,11-octadecadienoic acid methylester, linolenic acid methyl ester, phytol, linolenic acid, stigmast-5-en-3-ol (phytosterols) and β-amyrin in GC/MS analysis. Because the phenolic compounds can be degraded in GC/MS analysis, we performed HPLC analysis. Indeed, VOS has been reported to contain some phenolic compounds with anti-inflammatory activity such as (+)-catechin, (−)-epicatechin, proanthocyanidin A2 and cinnamtannin [17]. As shown in Fig. 2, VOS was analyzed to contain (+)-catechin, (−)-epicatechin and proanthocyanidin A2.

### Effect of the extracts from *V. oldhami* on LPS-mediated production of NO and PGE_2_ in RAW264 cells

**Fig. 3:**
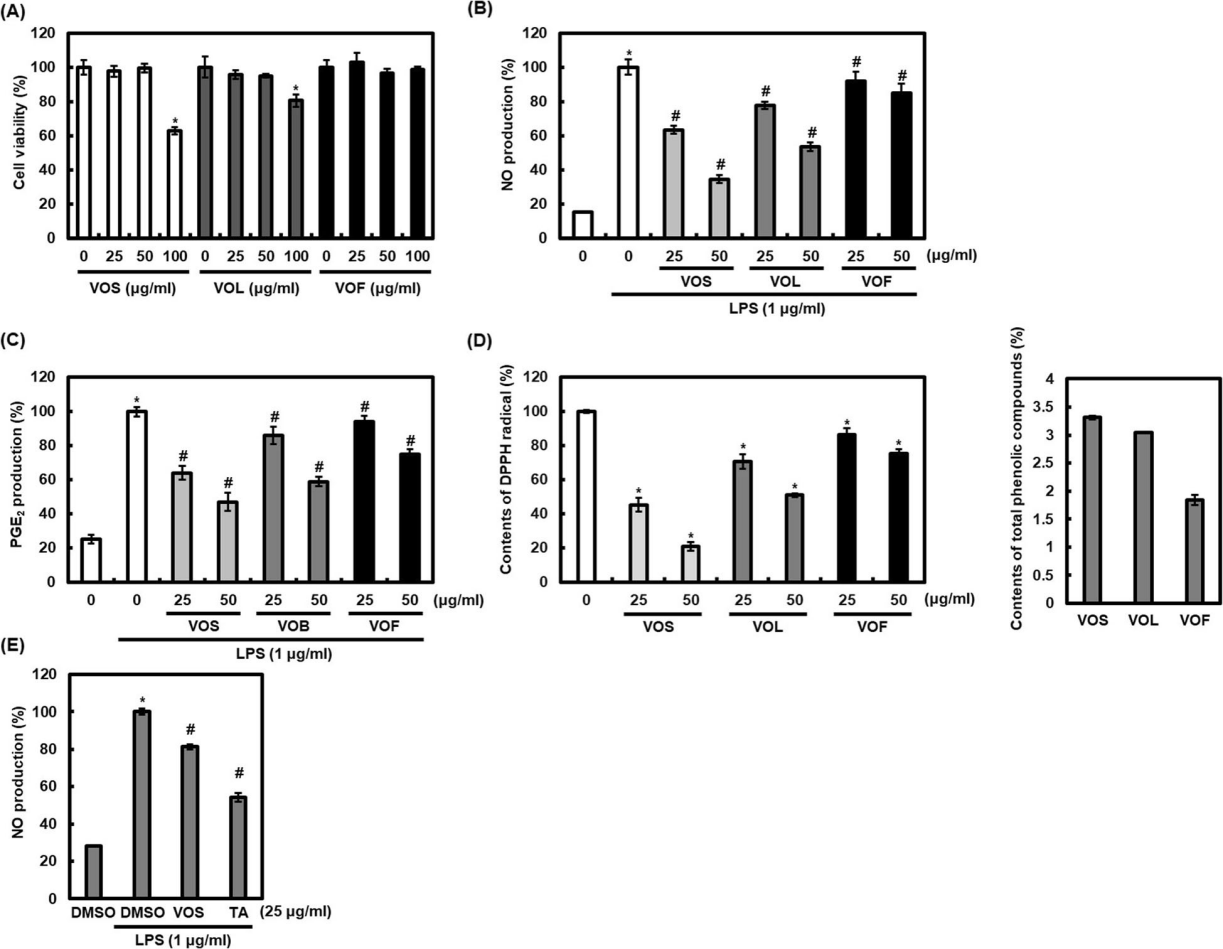
Inhibitory effect of the extracts from stems (VOS), leaves (VOL) and fruits (VOF) of *V. oldhami* against NO and PGE_2_ production in LPS-stimulated RAW264.7 cells. **a** RAW264.7 cells were treated with VOS, VOL or VOF for 24 h. Cell viability was measured using MTT assay. *P < 0.05 compared to the cells without the treatment. **b**, **c** RAW264.7 cells were pretreated with VOS, VOL or VOF for 6 h and then co-treated with LPS (1 μg/ml) for 18 h. NO and PGE_2_ production was measured by Griess assay and Prostaglandin E2 ELISA Kit, respectively. *P < 0.05 compared to the cells without the treatment, and ^#^*P* < 0.05 compared to the cells treated with LPS alone. **d** Antioxidant activity and contents of total phenolic compounds of VOS, VOL and VOF were analyzed as described in Materials and methods. **e** RAW264.7 cells were pretreated with VOS and TA for 6 h and then co-treated with LPS (1 μg/ml) for 18 h. NO production was measured by Griess assay. *P < 0.05 compared to the cells without the treatment, and ^#^*P* < 0.05 compared to the cells treated with LPS alone

To evaluate the cytotoxic effect of VOS, VOL and VOF, MTT assay was performed. As shown in Fig. 3a, VOS and VOL did not show cytotoxicity in RAW264.7 cells at 25 and 50 μg/ml, while 100 μg/ml of VOS and VOL reduced the viability of RAW264.7 cells. However, the reduction of cell viability by VOF (25–100 μg/ml) was not observed in RAW264.7 cells. Thus, 25 and 50 μg/ml of all extracts were selected for further study.

In order to compare the anti-inflammatory activity of *V. oldhami*, the inhibitory activities of the extracts from stems (VOS), leaves (VOL) and fruits (VOF) of *V. oldhami* on NO and PGE_2_ production were evaluated in LPS-stimulated RAW264.7 cells. As shown in Fig. 3b, Both VOS, VOL and VOF inhibited the overproduction of NO induced by LPS, but the NO inhibition potential of VOS was highest. In addition, the inhibition of PGE_2_ production by VOS, VOL and VOF was observed in LPS-stimulated RAW264.7 cells (Fig. 3c). The inhibitory effect of LPS-mediated PGE_2_ production was highest in VOS -treated RAW264.7 cells (Fig. 3c).

Because phenolic compounds with antioxidant activity have been reported to be closely related to anti-inflammatory activity [18, 19], the comparative studies of VOS, VOL and VOF for antioxidant activity and the content of total phenolic compounds were performed. In DPPH radical scavenging assay for evaluating antioxidant activity, the DPPH radical scavenging activity of VOS was higher than those of VOL and VOF. In addition, the content of total phenolic compounds from VOS was higher that of VOL and VOF (Fig. 3d). Thus, VOS was selected for further study. We also compared the inhibitory effect of VOS against NO production with TA as NSAIDs in LPS-stimulated RAW264.7 cells. As shown in Fig. 3e, VOS showed lower inhibitory activity against LPS-mediated NO production than TA.

### Effect of VOS on iNOS and COX-2 expression in LPS-stimulated RAW264.7 cells

**Fig. 4:**
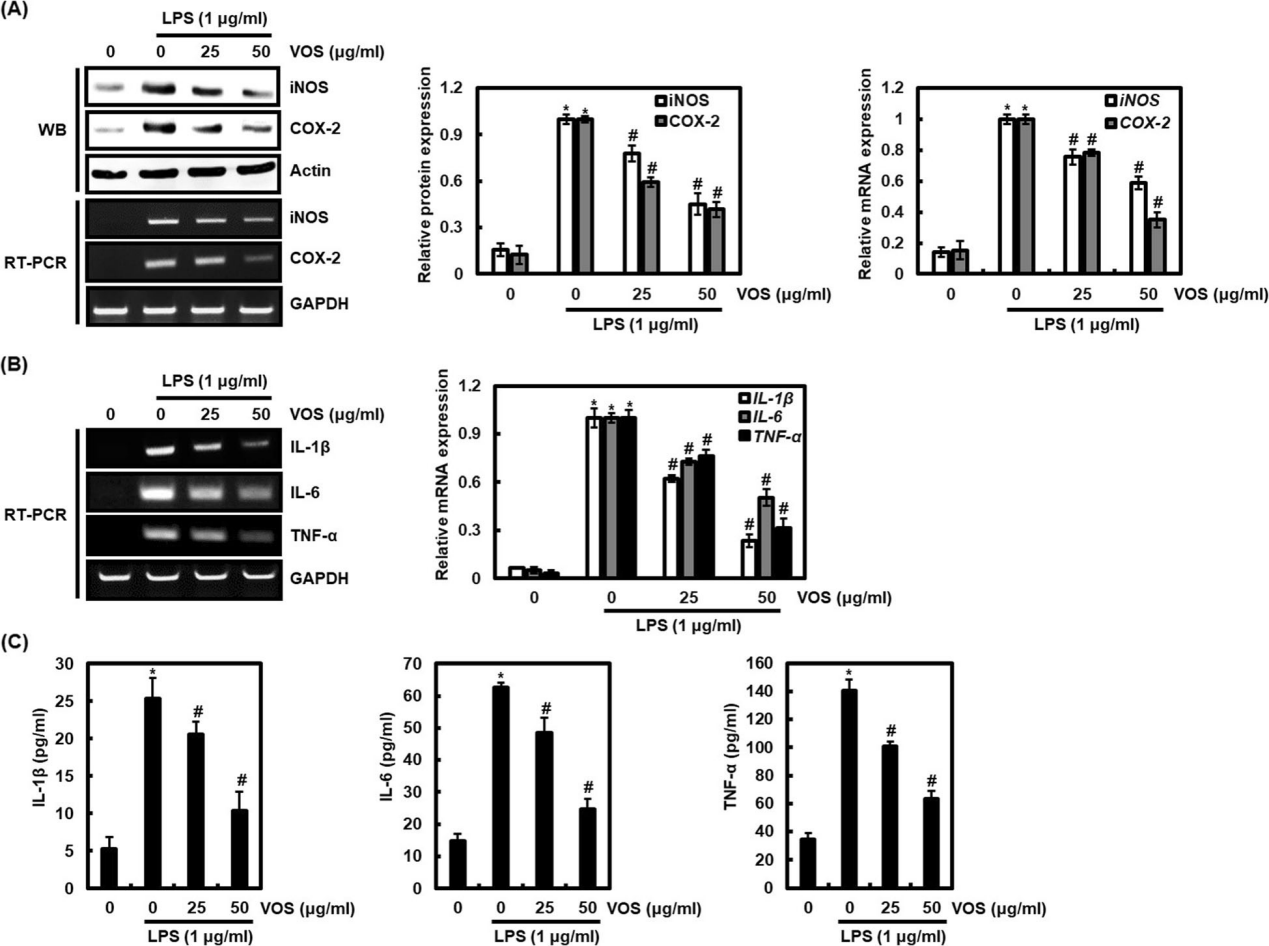
Effect of VOS on the expression of iNOS, COX-2, IL-1β, IL-6 and TNF-α in LPS-stimulated RAW264.7 cells. **a**, **b** RAW264.7 cells were pretreated with VOS for 6 h and then co-treated with LPS (1 μg/ml) for 18 h. For Western blot analysis, the cell lysates were subjected to SDS-PAGE and the Western blot was performed using antibodies against iNOS and COX-2. Actin was used as internal control for Western blot analysis. For RT-PCR analysis, total RNA was prepared. GAPDH was used as internal control for RT-PCR. The density of mRNA and protein bands was calculated using the software UN-SCAN-IT gel version 5.1 (Silk Scientific Inc. Orem, UT, USA). *P < 0.05 compared to the cells without the treatment, and ^*#*^*P* < 0.05 compared to the cells treated with LPS alone. **c** RAW264.7 cells were pretreated with VOS for 6 h and then co-treated with LPS (1 μg/ml) for 18 h. IL-1β, IL-6 and TNF-α were measured using ELISA Kit

Because the expression of iNOS is closely related to the production of NO [7, 8], we evaluated whether decreased NO production by VOS is due to the downregulation of iNOS expression. As a result, VOS inhibited iNOS expression at both mRNA and protein level in LPS-stimulated RAW264.7 cells. (Fig. 4a). These results indicate that NO production reduced by VOS may be due to the inhibition of iNOS expression.

In addition, PGE_2_, which is overproduced by COX-2, induces the tissue damage by excessive inflammatory response [20], so that the inhibition of abnormal COX-2 expression is known to be an important target for inhibiting inflammatory diseases [21]. Thus, we evaluated whether the inhibition of PGE_2_ production by VOS results from the inhibition of COX-2 expression. As shown in Fig. 4a, VOS dose-dependently inhibited COX-2 overexpression induced by LPS at both mRNA and protein level in RAW264.7 cells. These results indicates that VOS -mediated inhibition of COX-2 expression may contribute to the inhibition of PGE_2_ production.

### Effect of VOS on the expression of pro-inflammatory cytokines such as IL-1β, IL-6 and TNF-α in LPS-stimulated RAW264.7 cells

To evaluate whether VOS affects the expression of pro-inflammatory cytokines such as IL-1β, IL-6 and TNF-α, RAW264.7 cells were treated with VOS in presence of LPS. As shown in Fig. 4b, VOS significantly inhibited LPS-mediated overexpression of pro-inflammatory cytokines such as IL-1β, IL-6 and TNF-α in RAW264.7 cells. We also confirmed the inhibitory effect of VOS against LPS-induced overexpression of pro-inflammatory cytokines such as IL-1β, IL-6 and TNF-α using ELISA assay in RAW264.7 cells. As shown in Fig. 4c, the overproduction of IL-1β, IL-6 and TNF-α induced by LPS was decreased by the treatment of VOS in a dose-dependent manner.

### Effect of VOS on the osteoclastogenesis in RANKL-stimulated RAW264.7 cells

**Fig. 5:**
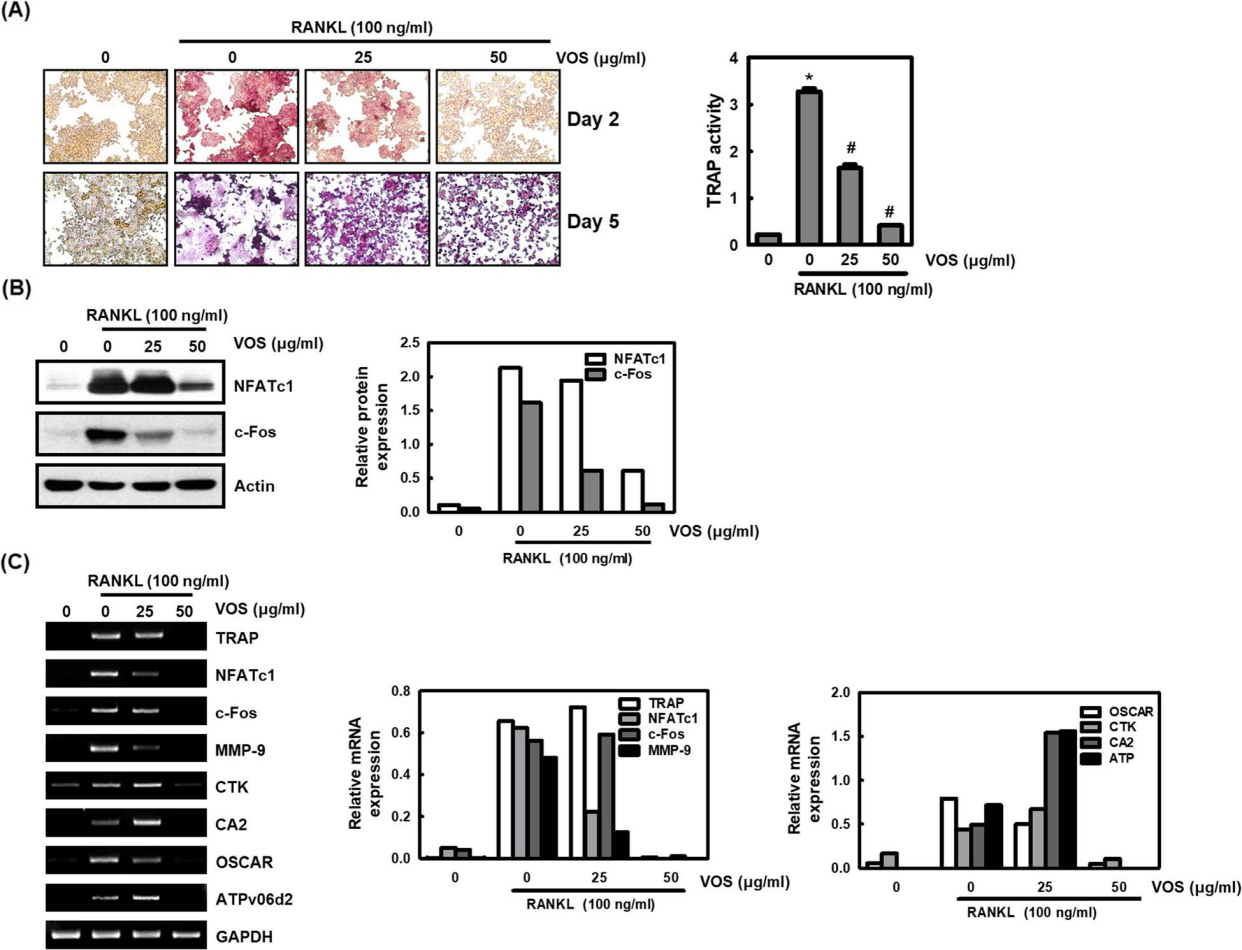
Effect of VOS on osteoclast differentiation. **a** Effect of VOS on osteoclastogenesis and resorptive activity. The cells were stained with the TRAP assay kit, and media were collected for the TRAP activity. TRAP-positive multinuclear cells were captured using an inverted microscope (100×, Scale bars: 200 mm). The media were measured for TRAP activity by an ELISA reader. **b** Effect of VOS on the activation of NFATc1 and c-Fos by RANKL. NFATc1 and c-Fos protein expressions were determined by western blot. Expressions of NFATc1 and c-Fos were normalized to actin. **c** Effects of VOS on the mRNA expression of osteoclastogenesis marker genes by RANKL. The mRNA expressions were detected by RT-PCR and normalized to GAPDH. Columns and error bars represent the mean ± S.D. of three independent experiments. The density of mRNA bands was calculated using the software UN-SCAN-IT gel version 5.1 (Silk Scientific Inc. Orem, UT, USA). *P < 0.05 compared to the cells without the treatment, and ^*#*^*P* < 0.05 compared to the cells treated with RANKL alone

Among various pro-inflammatory mediators, NO and PGE_2_ promote osteoclast-induced bone resorption [5, 22, 23]. To measure the effect of VOS on osteoclast formation using the murine monocyte/macrophage cell line RAW 264.7, RANKL (100 ng/ml) was used to induce TRAP-positive multinucleated osteoclast differentiation in RAW 264.7 cells. VOS had inhibitory effects on TRAP-positive cells in a dose-dependent manner (Fig. 5a). Furthermore, VOS also had an inhibitory effect on the TRAP activity (Fig. 5a). These data were consistent with the inhibitory effects on osteoclast formation. The effect of VOS on essential osteoclast differentiation indicators such as NFATc1 and c-Fos was investigated. NFATc1 is controlled by c-Fos as a master transcription factor for osteoclast differentiation. VOS had significant inhibitory effects on the expression of NFATc1 and c-Fos protein expression (Fig. 5b). We also examined whether VOS regulates the expression of osteoclastogenesis markers by inhibiting the NFATc1/c-Fos signaling pathways. VOS suppressed mRNA expression of osteoclast-related genes such as TRAP, CTK, OSCAR, ATP6v0d2, and CA2 controlled by NFATc1/c-Fos (Fig. 5c). The expression of all mRNA was significantly inhibited by VOS (Fig. 5c).

### Effect of VOS on NF-κB signaling activation in LPS-stimulated RAW264.7 cells

**Fig. 6:**
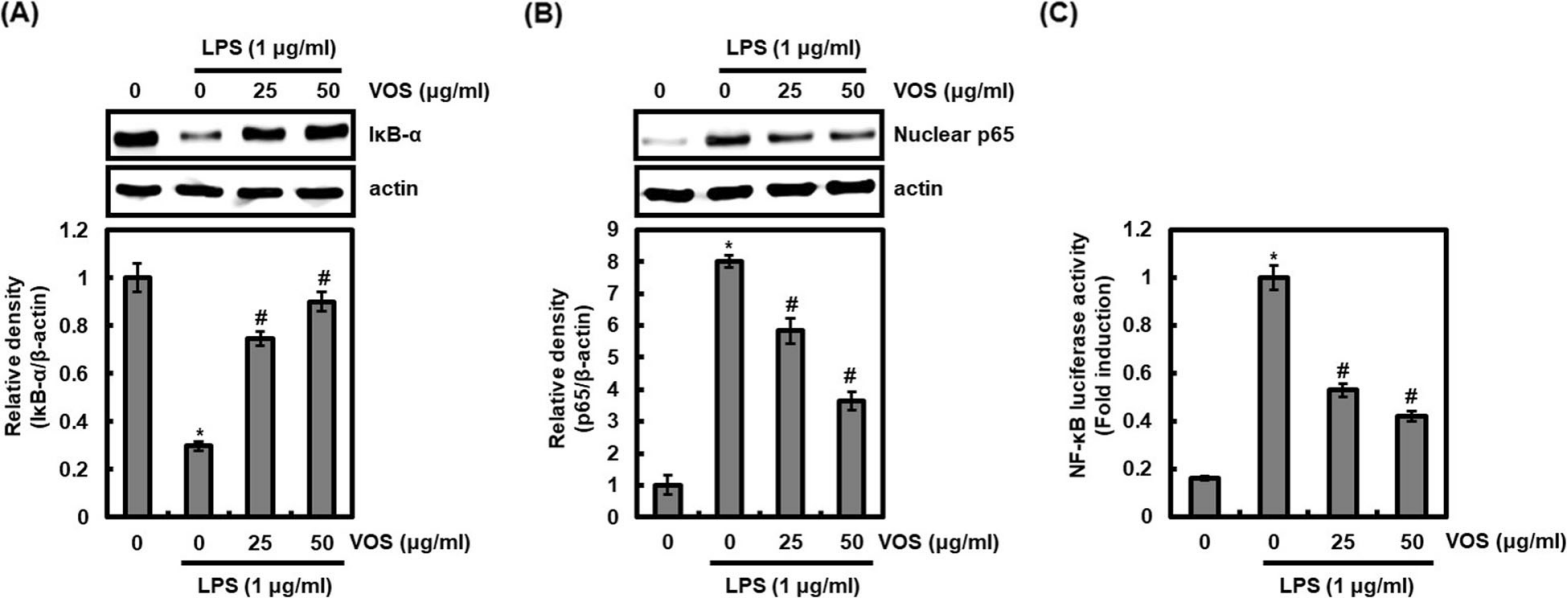
Effect of VOS on NF-κB signaling activation. **a** RAW264.7 cells were pretreated with VOS for 6 h and then co-treated with LPS (1 μg/ml) for 20 min. **b** RAW264.7 cells were pretreated with VOS for 6 h and then co-treated with LPS (1 μg/ml) for 30 min. After the treatment, the nucleus fraction was prepared. For Western blot analysis, the cell lysates were subjected to SDS-PAGE and the Western blot was performed using antibodies against IκB-α and p65. Actin was used as internal control for Western blot analysis. *P < 0.05 compared to the cells without the treatment, and ^*#*^*P* < 0.05 compared to the cells treated with LPS alone. **c** RAW264.7 cells were co-transfected with NF-κB luciferase constructs and pRL-null. The cells were pretreated with VOS for 6 h and then co-treated with LPS (1 μg/ml) for 18 h. Luciferase activity for NF-κB was measured as a ratio of firefly luciferase signal/renilla luciferase signal using a dual luciferase assay kit. The density of Western blot bands was calculated using the software UN-SCAN-IT gel version 5.1 (Silk Scientific Inc. Orem, UT, USA).*P < 0.05 compared to the cells without the treatment, and ^*#*^*P* < 0.05 compared to the cells treated with LPS alone

Because NF-κB is known to be the major signal transduction pathway in inflammatory response and osteoclastogenesis [24], inhibition of NF-κB signaling by VOS was evaluated by inhibition of IκB-α degradation and nuclear translocation of p65. As shown in Fig. 6a, the treatment of LPS alone resulted in the degradation of IκB-α, but VOS inhibited the degradation of IκB-α induced by LPS in RAW264.7 cells. NF-κB released by IκB-α degradation translocates to the nucleus and binds to genes involved in pro-inflammatory mediators and osteoclastogenesis. Thus, it was evaluated whether the inhibition of IκB-α degradation by VOS induces the inhibition of NF-κB nuclear translocation. As shown in Fig. 6b, LPS induced p65 nuclear accumulation, but the presence of VOS suppressed LPS-mediated p65 nuclear accumulation, which resulted in the inhibition of NF-κB activation (Fig. 6c). These results indicate that VOS may inhibit NF-κB activation by blocking IκB-α degradation and subsequent p65 nuclear translocation in the inflammatory responses.

### Effect of VOS on MAPK/ATF2 signaling activation in LPS-stimulated RAW264.7 cells

**Fig. 7:**
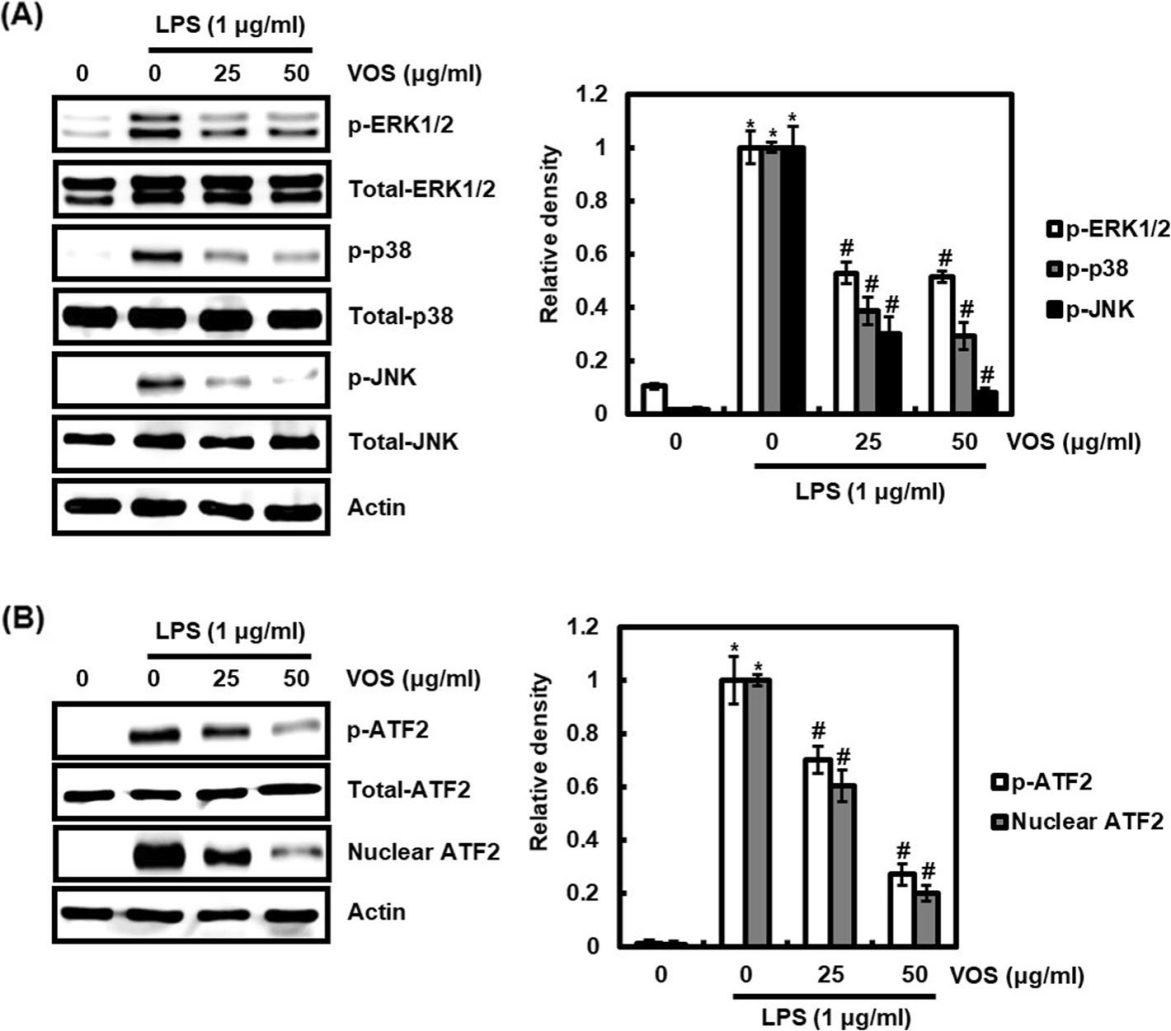
Effect of VOS on MAPK/ATF2 signaling activation. **a** RAW264.7 cells were pretreated with VOS for 6 h and then co-treated with LPS (1 μg/ml) for 20 min. **b** RAW264.7 cells were pretreated with VOS for 6 h and then co-treated with LPS (1 μg/ml) for 30 min. After the treatment, the nucleus fraction was prepared. For Western blot analysis, the cell lysates were subjected to SDS-PAGE and the Western blot was performed using antibodies against p-ERK1/2, p-p38, p-JNK p-ATF2 and ATF2. Total-ERK1/2, total-p38 and total-JNK and actin were used as internal control for Western blot analysis. The density of Western blot bands was calculated using the software UN-SCAN-IT gel version 5.1 (Silk Scientific Inc. Orem, UT, USA). *P < 0.05 compared to the cells without the treatment, and ^#^*P* < 0.05 compared to the cells treated with LPS alone

MAPK, composed of ERK1/2, p38 and JNK is also a major signaling pathway in the inflammatory response [9], and activation of MAPK signaling promotes osteoclastogenesis [23, 25]. Thus, we assessed whether VOS inhibits MAPK signaling through the inhibition of phosphorylation of ERK1/2, p38 and JNK. As shown in Fig. 7a, LPS induced the phosphorylation of ERK1/2, p38 and JNK, but the presence of VOS suppressed the phosphorylation of ERK1/2, p38 and JNK in LPS-stimulated RAW264.7 cells, which indicates that VOS may inhibit MAPK activation. MAPK activation can induce nuclear accumulation of ATF2 through ATF2 phosphorylation, and this signaling promotes expression of pro-inflammatory mediators [26] and osteoclastogenesis [27, 28]. As shown in Fig. 7b, VOS dose-dependently inhibited LPS-induced phosphorylation of ATF2 and reduced the nuclear accumulation of ATF2. These results indicate that VOS may inhibit MAPK/ATF2 signaling activation.

## Discussion

Since inflammatory diseases are considered to be one of the major health problems, the development of anti-inflammatory drugs for the treatment of inflammatory diseases has been longstanding. Currently, non-steroid anti-inflammatory drugs (NSAIDs) have been prescribed for the treatment of inflammatory diseases, but the long-term use of NSAIDs is known to cause serious side effects [29]. Thus, the importance of searching for anti-inflammatory candidates with low side effects has been emphasized. In this study, we demonstrated that stem extracts from *V. oldhami* (VOS) inhibits LPS-stimulated inflammatory response in RAW264.7 cells.

Overproduced nitric oxide (NO) by inducible nitric oxide synthase (iNOS) and interleukin 1β (IL-1β) has been reported to be associated with the onset of chronic diseases [8, 30, 31]. NO can promote osteoclast formation by inducing cell fusion and increasing actin remodeling in mononuclear pre-osteoclast, which eventually results in fusion and formation of multinucleated osteoclasts [22, 32]. In addition, NO produced by iNOS activates osteoclast, resulting in bone loss [33]. IL-1β involved in NO production has been reported to directly or indirectly cause osteolysis [34]. It is known that increased prostaglandin E_2_ (PGE_2_) produced by cyclooxygenase-2 (COX-2) in excessive inflammation also causes inflammatory-bone resorption [35], so that inhibition of COX-2 expression can suppress osteoclast-induced bone loss [36, 37]. Therefore, inhibition of NO production by blocking iNOS and IL-1β expression and PGE_2_ production by blocking COX-2 expression may be a useful clinic strategy for treating inflammatory osteoporosis.

In this study, we observed that VOS inhibited LPS-induced NO and PGE_2_ production through inhibition of iNOS and IL-1β, and COX-2 expression, respectively. In addition, VOS blocked IL-6 and TNF-α expression. In order to confirm the degree of anti-inflammatory activity of VOS, we compared the inhibitory effect of VOS against LPS-induced overproduction of NO with tolfenamic acid (TA) as one of non-steroidal anti-inflammatory drugs. Although VOS had a lower inhibitory activity against LPS-induced NO production than TA, VOS is considered to be a potential source for the development of anti-inflammatory drugs because VOS is a crude extract.

To demonstrate the mechanism of osteoclast suppression of VOS, a RANKL-induced RAW264.7 cells were used [38]. TRAP secreted only by osteoclast has been considered as a phenotype of osteoclasts [39]. In the present study, VOS inhibited osteoclast differentiation and its activity. Previous studies have demonstrated that NFATc1 and c-Fos are the master regulator in osteoclastogenesis [40, 41]. In addition, overexpression of NFATc1 and c-Fos by RANKL induces differentiation of osteoclast precursor cells into osteoclasts [40, 42]. We observed that VOS inhibited the expression of NFATc1 and c-Fos. Additional, NFATc1 and c-Fos regulate various markers involved in osteoclast such as MMP-9, CTK and CA2. These genes play an important role in the degradation and resorption of the bone matrix [43]. CA2 is placed on the bone matrix and acidifies the bone surface [44]. After that, bone resorption markers such as MMP-9, CTK lead to absorb. OSCAR is related to osteoclast differentiation and bone homeostasis [45]. ATP6v0d2 is an indicator of cell fusion in osteoclastogenesis and important constituent of osteoclast-related proton pump that controls acidification in matrix of bone [46]. In the present study, VOS inhibited various genes related to osteoclast differentiation. These results indicated that VOS has an inhibitory effect on osteoclast differentiation by suppressing expression of osteoclastogenesis marker genes.

Abnormal activation of nuclear factor kappaB (NF-κB) signaling in excessive inflammatory responses is closely related to onset of various inflammatory diseases such as rheumatoid arthritis, atherosclerosis, chronic obstructive pulmonary disease, asthma, inflammatory bowel disease and ulcerative colitis [47, 48], and also induces osteoclast formation by increasing expression of NFATc1 [49]. Thus, inhibition of NF-κB signaling activation may provide an effective approach to inhibit osteoclast-induced bone resorption by excessive inflammatory responses. In current study, the inhibition of LPS-induced NF-κB signaling activation by VOS was confirmed by the inhibition of VOS on IκB-α degradation, p65 nuclear accumulation and NF-κB luciferase activation. These results indicate that VOS may inhibit the abnormal inflammatory response and inflammation-induced osteoclastogenesis via NF-κB signaling.

There is growing evidence that mitogen-activated protein kinases (MAPK), known as excessive inflammatory signaling, also plays a positive role in osteoclastogenesis [50]. Indeed, the inhibition of ERK1/2, p38 and JNK is known to inhibit the differentiation of osteoclast-precursor cells into osteoclast [23]. Activating transcription factor 2 (ATF2) activation by its phosphorylation and subsequent nuclear accumulation has been reported to be involved in MAPK signaling-induced production of the inflammatory mediators [25]. In addition, ATF2, which is activated by MAPK signaling, has been established to be involved in osteoclast differentiation [26, 27]. Luteolin, a flavonoid compound, has been reported to inhibit osteoclast differentiation through inhibition of ATF2 activation.

In GC/MS and HPLC analysis, we observed that VOS contained several compounds with anti-inflammatory activity such as 4-((1E)-3-Hydroxy-1-propenyl)-2-methoxyphenol [51], methyl palmitate [52], n-hexadecanoic acid [53], sinapyl alcohol [54], phytol [55], linolenic acid [56], stigmast-5-en-3-ol (phytosterols) [57], β-amyrin [58], (+)-catechin [59], (−)-epicatechin [59] and proanthocyanidin [60]. Although various compounds with anti-inflammatory activity were analyzed from VOS, it is necessary to investigate which compounds affect the anti-inflammatory activity of VOS through activation tracing separation.

In this study, we confirmed that VOS inhibits MAPK activation through blocking the phosphorylation of ERK1/2, p38 and JNK, and MAPK-induced phosphorylation and nuclear accumulation of ATF2. These results indicate that VOS may inhibit the abnormal inflammatory response and inflammation-induced osteoclastogenesis via MAPK/ATF2 signaling.

## Conclusion

Taken together, these results show that VOS inhibits the expression of pro-inflammatory mediators and osteoclastogenesis-related factors through suppressing the NF-κB and MAPK/ATF2 signaling activation. These results will provide the potential value for the development of anti-inflammatory and anti-osteoporosis drug using VOS.

## Data Availability

The datasets used and/or analyzed during the current study available from the corresponding author on reasonable request.

## Abbreviations

CAII: Carbonic anhydrase II
COX-2: Cyclooxygenase-2
IL-1β: Interleukin-1β
iNOS: Inducible nitric oxide synthase
LPS: Lipopolysaccharide
MTT: 3-(4,5-dimethylthiazol-2-yl)-2,5-diphenyltetrazolium bromide
NFATc1: Nuclear factor of activated T-cells, cytoplasmic 1
NO: Nitric oxide
PGE_2_: Prostaglandin E_2_
TRAP: Tartrate-resistant acid phosphatase
VOF: Fruit extracts from *Vaccinium oldhamii*
VOL: Leave extracts from *Vaccinium oldhamii*
VOS: Stem extracts from *Vaccinium oldhamii*

## References

[1] Hotamisligil GS, Erbay E (2008). Nutrient sensing and inflammation in metabolic diseases. Nat Rev Immunol.

[2] Libby P (2006). Inflammation and cardiovascular disease mechanisms. Am J Clin Nutr.

[3] Wyss-Coray T, Mucke L (2002). Inflammation in neurodegenerative disease-a double-edged sword. Neuron.

[4] Trinchieri G (2012). Cancer and inflammation: an old intuition with rapidly evolving new concepts. Ann Rev Immunol.

[5] Hou GQ, Guo C, Song GH, Fang N, Fan WJ, Chen XD, Yuan L, Wang ZQ (2013). Lipopolysaccharide (LPS) promotes osteoclast differentiation and activation by enhancing the MAPK pathway and COX-2 expression in RAW264.7 cells. Int. J Mol Med.

[6] Sharma JN, Al-Omran A, Parvathy SS (2007). Role of nitric oxide in inflammatory diseases. Inflammopharmacology.

[7] Coleman JW (2001). Nitric oxide in immunity and inflammation. Int Immunopharmacol.

[8] Kitade H, Sakitani K, Inoue K, Masu Y, Kawada N, Hiramatsu Y, Kamiyama Y, Okumura T, Ito S (2003). Interleukin 1b markedly stimulates nitric oxide formation in the absence of other cytokines or lipopolysaccharide in primary cultured rat hepatocytes but not in kupffer cells. Hepatology.

[9] Nakagawa H, Maeda S (2012). Molecular mechanisms of liver injury and hepatocarcinogenesis: focusing on the role of stress-activated MAPK. Pathol Res Int.

[10] Seibert K, Masferrer JL (1994). Role of inducible cyclooxygenase (COX-2) in inflammation. Receptor.

[11] Ginaldi L, Di Benedetto MC, De Martinis M (2005). Osteoporosis, inflammation and ageing. Immun Ageing.

[12] Lee JH, Lee KT, Yang JH, Baek NI, Kim DK (2004). Acetylcholinesterase inhibitors from the twigs of *Vaccinium oldhami* Miquel. Arch Pharm Res.

[13] Hirotoshi T, Hisato K, Ryoko KT, Kazuo N, Masao Y, Haruki K, Chizyko Y (2013). Antioxidant activities and anti-cancer cell proliferation properties of Natsuhaze (*Vaccinium oldhamii* Miq.), Shashanbo (*V. bracteatum* Thunb.) and blueberry cultivars. Plants.

[14] Oh SJ, Koh SC (2009). Screening of antioxidative activity and a-amylase inhibitory activity in angiosperm plants native to Jeju Island. Korean J Plant Res.

[15] Baba T, Hirose D, Sasaki N, Watanabe N, Kobayashi N, Kurashige Y, Karimi F, Ban T (2016). Mycorrhizal formation and diversity of Endophytic Fungi in hair roots of V*accinium oldhamii* Miq. In Japan. Microbes Environ.

[16] Yang EJ, Yim EY, Song G, Kim GO, Hyun CG (2009). Inhibition of nitric oxide production in lipopolysaccharide-activated RAW 264.7 macrophages by Jeju plant extracts. Interdiscip. Toxicol..

[17] Park HW, Kim DK (2005). Tannin components from the twigs of *Vaccinium oldhami* Miquel. Kor J Pharmacogn.

[18] Arulselvan P, Fard MT, Tan WS, Gothai S, Fakurazi S, Norhaizan ME, Kumar SS (2016). Role of antioxidants and natural products in inflammation. Oxidative Med Cell Longev.

[19] Ravipati AS, Zhang L, Koyyalamudi SR, Jeong SC, Reddy N, Bartlett J, Smith PT, Shanmugam K, Münch G, Wu MJ, Satyanarayanan M, Vysetti B (2012). Antioxidant and anti-inflammatory activities of selected Chinese medicinal plants and their relation with antioxidant content. BMC Complement Altern Med.

[20] Blotman F, Poubelle P, Chaintreuil J, Damon M, Flandre O, Crastes de Paulet A, Simon L (1982). Mononuclear phagocytes, prostanoids and rheumatoid arthritis. Int J Immunopharmacol.

[21] Guan F, Wang H, Shan Y, Chen Y, Wang M, Wang Q, Yin M, Zhao Y, Feng X, Zhang J (2014). Inhibition of COX-2 and PGE2 in LPS-stimulated RAW264.7 cells by lonimacranthoide VI, a chlorogenic acid ester saponin. Biomed. Rep..

[22] Blackwell KA, Raisz LG, Pilbeam CC (2010). Prostaglandins in bone: bad cop, good cop?. Trends Endocrinol Metab.

[23] Park EJ, Kim SA, Choi YM, Kwon HK, Shim W, Lee G, Choi S (2011). Capric acid inhibits NO production and STAT3 activation during LPS-induced osteoclastogenesis. PLoS One.

[24] Li L, Sapkota M, Kim SW, Soh Y (2016). Herbacetin inhibits RANKL-mediated osteoclastogenesis in vitro and prevents inflammatory bone loss in vivo. Eur J Pharmacol.

[25] Takayanagi H (2007). Osteoimmunology: shared mechanisms and crosstalk between the immune and bone systems. Nat. Rev. Immunol..

[26] Yu T, Li YJ, Bian AH, Zuo HB, Zhu TW, Ji SX, Kong F, Yin DQ, Wang CB, Wang ZF, Wang HQ, Yang Y, Yoo BC, Cho JY (2014). The regulatory role of activating transcription factor 2 in inflammation. Mediat Inflamm.

[27] Inoue K, Imai Y (2014). Identification of novel transcription factors in osteoclast differentiation using genome-wide analysis of open chromatin determined by DNase-seq. J Bone Miner Res.

[28] Lee JW, Ahn JY, Hasegawa S, Cha BY, Yonezawa T, Nagai K, Seo HJ, Jeon WB, Woo JT (2009). Inhibitory effect of luteolin on osteoclast differentiation and function. Cytotechnology.

[29] Sostres C, Gargallo CJ, Arroyo MT, Lanas A (2010). Adverse effects of non-steroidal anti-inflammatory drugs (NSAIDs, aspirin and coxibs) on upper gastrointestinal tract. Best Pract Res Clin Gastroenterol.

[30] Park JY, Cho HY, Kim JK, Noh KH, Yang JR, Ahn JM, Lee MO, Song YS (2005). Chlorella dichloromethane extract ameliorates NO production and iNOS expression through the down-regulation of NF kappa B activity mediated by suppressed oxidative stress in RAW 264.7 macrophages. Int J Clin Chem.

[31] Li L, Sapkota M, Kim SW, Soh Y (2015). Herbacetin inhibits inducible nitric oxide synthase via JNK and nuclear factor-kappaB in LPS-stimulated RAW264.7 cells. Eur J Pharmacol.

[32] Nilforoushan D, Gramoun A, Glogauer M, Manolson MF (2009). Nitric oxide enhances osteoclastogenesis possibly by mediating cell fusion. Nitric Oxide.

[33] Herrera BS, Martins-Porto R, Maia-Dantas A, Campi P, Spolidorio LC, Costa SK, Van Dyke TE, Gyurko R, Muscara MN (2011). iNOS-derived nitric oxide stimulates osteoclast activity and alveolar bone loss in ligature-induced periodontitis in rats. J Periodontol.

[34] Zhang Y, Yan M, Yu QF, Yang PF, Zhang HD, Sun YH, Zhang ZF, Gao YF (2016). Puerarin prevents LPS-induced osteoclast formation and bone loss via inhibition of Akt activation. Biol Pharm Bull.

[35] Coon D, Gulati A, Cowan C, He J (2007). The role of cyclooxygenase-2 (COX-2) in inflammatory bone resorption. J Endod.

[36] Ono K, Akatsu T, Kugai N, Pilbeam CC, Raisz LG (2003). The effect of deletion of cyclooxygenase-2, prostaglandin receptor EP2, or EP4 in bone marrow cells on osteoclasts induced by mouse mammary cancer cell lines. Bone.

[37] Kellinsalmi M, Parikka V, Risteli J, Hentunen T, Leskela HV, Lehtonen S, Selander K, Vaananen K, Lehenkari P (2007). Inhibition of cyclooxygenase-2 down-regulates osteoclast and osteoblast differentiation and favours adipocyte formation in vitro. Eur J Pharmacol.

[38] Su H, Lacey DL, Dunstan CR, Solovyev I, Colombero A, Timms E, Tan HL, Elliott G, Kelley MJ, Sarosi I, Wang L, Xia XZ, Elliott R, Chiu L, Black T, Scully S, Capparelli C, Morony S, Shimamoto G, Bass MB, Boyle WJ (1999). Tumor necrosis factor receptor family member RANK mediates osteoclast differentiation and activation induced by osteoprotegerin ligand. Proc Natl Acad Sci U S A.

[39] Habermann B, Eberhardt C, Feld M, Zichner L, Kurth AA (2007). Tartrate-resistant acid phosphatase 5b (TRAP 5b) as a marker of osteoclast activity in the early phase after cementless total hip replacement. Acta Orthop.

[40] Zhao Q, Wang X, Liu Y, He A, Jia R (2010). NFATc1: functions in osteoclasts. Int J Biochem Cell Biol.

[41] Grigoriadis AE, Wang ZQ, Cecchini MG, Hofstetter W, Felix R, Fleisch HA, Wagner EF (1994). C-Fos: a key regulator of osteoclast-macrophage lineage determination and bone remodeling. Science.

[42] Wang ZQ, Ovitt C, Grigoriadis AE, Mohle-Steinlein U, Ruther U, Wagner EF (1992). Bone and haematopoietic defects in mice lacking c-fos. Nature.

[43] Fujisaki K, Tanabe N, Suzuki N, Kawato T, Takeichi O, Tsuzukibashi O, Makimura M, Ito K, Maeno M (2007). Receptor activator of NF-kappaB ligand induces the expression of carbonic anhydrase II, cathepsin K, and matrix metalloproteinase-9 in osteoclast precursor RAW264.7 cells. Life Sci.

[44] David JP, Rincon M, Neff L, Horne WC, Baron R (2001). Carbonic anhydrase II is an AP-1 target gene in osteoclasts. J Cell Physiol.

[45] Nemeth K, Schoppet M, Al-Fakhri N, Helas S, Jessberger R, Hofbauer LC, Goettsch C (2011). The role of osteoclast-associated receptor in osteoimmunology. J Immunol.

[46] Wu H, Xu G, Li YP (2009). Atp6v0d2 is an essential component of the osteoclast-specific proton pump that mediates extracellular acidification in bone resorption. J Bone Miner Res.

[47] Tak PP, Firestein GS (2001). NF-kappaB: a key role in inflammatory diseases. J Clin Invest.

[48] Lawrence T (2009). The nuclear factor NF-kappaB pathway in inflammation. Cold Spring Harb Perspect Biol.

[49] Abu-Amer Y (2013). NF-kappaB signaling and bone resorption. Osteoporos Int.

[50] Cheng B, Li J, Du J, Lv X, Weng L, Ling C (2012). Ginsenoside Rb1 inhibits osteoclastogenesis by modulating NF-kappaB and MAPKs pathways. Food Chem Toxicol.

[51] Dandekar R, Fegade B, Bhaskar V (2015). GC-MS analysis of phytoconstituents in alcohol extract of *Epiphyllum oxypetalum* leaves. J Pharmacogn Phytochem.

[52] Saeed NM, El-Demerdash E, Abdel-Rahman HM, Algandaby MM, Al-Abbasi FA, Abdel-Naim AB (2012). Anti-inflammatory activity of methyl palmitate and ethyl palmitate in different experimental rat models. Toxicol Appl Pharmacol.

[53] Aparna V, Dileep KV, Mandal PK, Karthe P, Sadasivan C, Haridas M (2012). Anti-inflammatory property of n-hexadecanoic acid: structural evidence and kinetic assessment. Chem Biol Drug Des.

[54] Choi J, Shin KM, Park HJ, Jung HJ, Kim HJ, Lee YS, Rew JH, Lee KT (2004). Anti-inflammatory and antinociceptive effects of sinapyl alcohol and its glucoside syringin. Planta Med.

[55] Silva RO, Sousa FB, Damasceno SR, Carvalho NS, Silva VG, Oliveira FR, Sousa DP, Aragão KS, Barbosa AL, Freitas RM, Medeiros JV (2014). Phytol, a diterpene alcohol, inhibits the inflammatory response by reducing cytokine production and oxidative stress. Fundam Clin Pharmacol.

[56] Reifen R, Karlinsky A, Stark AH, Berkovich Z, Nyska A (2015). α-Linolenic acid (ALA) is an anti-inflammatory agent in inflammatory bowel disease. J Nutr Biochem.

[57] Aldini R, Micucci M, Cevenini M, Fato R, Bergamini C, Nanni C, Cont M, Camborata C, Spinozzi S, Montagnani M, Roda G, D’Errico-Grigioni A, Rosini F, Roda A, Mazzella G, Chiarini A, Budriesi R (2014). Antiinflammatory effect of phytosterols in experimental murine colitis model: prevention, induction, remission study. PLoS One.

[58] Krishnan K, Mathew LE, Vijayalakshmi NR, Helen A (2014). Anti-inflammatory potential of beta-amyrin, a triterpenoid isolated from Costus igneus. Inflammopharmacology.

[59] Chang HH, Chien CY, Chen KH, Huang SC, Chien CT (2017). Catechins blunt the effects of oxLDL and its primary metabolite phosphatidylcholine hydroperoxide on endothelial dysfunction through inhibition of oxidative stress and restoration of eNOS in rats. Kidney Blood Press Res.

[60] Zhang M, Wu Q, Chen Y, Duan M, Tian G, Deng X, Sun Y, Zhou T, Zhang G, Chen W, Chen J (2018). Inhibition of proanthocyanidin A2 on porcine reproductive and respiratory syndrome virus replication in vitro. PLoS One.

